# Ketone Bodies in Diabetes Mellitus: Friend or Foe?

**DOI:** 10.3390/nu15204383

**Published:** 2023-10-16

**Authors:** Stavroula Veneti, Maria G. Grammatikopoulou, Evangelia Kintiraki, Gesthimani Mintziori, Dimitrios G. Goulis

**Affiliations:** 1 Unit of Reproductive Endocrinology, 1st Department of Obstetrics and Gynecology, Medical School, Aristotle University of Thessaloniki, GR-54124 Thessaloniki, Greece; stavroulav2@hotmail.com (S.V.); evakintiraki@gmail.com (E.K.);; 2 Unit of Immunonutrition and Clinical Nutrition, Department of Rheumatology and Clinical Immunology, Faculty of Medicine, School of Health Sciences, University of Thessaly, Biopolis, GR-41110 Larissa, Greece

**Keywords:** low-carbohydrate diet, low glycemic index, middle-chain triglycerides, carbohydrate restriction, weight loss, obesity, prediabetes, β-hydroxybutyrate, acetoacetate

## Abstract

In glucose-deprived conditions, ketone bodies are produced by the liver mitochondria, through the catabolism of fatty acids, and are used peripherally, as an alternative energy source. Ketones are produced in the body under normal conditions, including during pregnancy and the neonatal period, when following a ketogenic diet (KD), fasting, or exercising. Additionally, ketone synthesis is also augmented under pathological conditions, including cases of diabetic ketoacidosis (DKA), alcoholism, and several metabolic disorders. Nonetheless, diet is the main regulator of total body ketone concentrations. The KDs are mimicking the fasting state, altering the default metabolism towards the use of ketones as the primary fuel source. Recently, KD has gained recognition as a medical nutrition therapy for a plethora of metabolic conditions, including obesity and diabetes mellitus (DM). The present review aims to discuss the role of ketones, KDs, ketonemia, and ketonuria in DM, presenting all the available new evidence in a comprehensive manner.

## 1. Introduction

Diabetes mellitus (DM) is a leading cause of morbidity, disability, and premature mortality globally, multiplying the risk of developing cardiovascular disease (CVD) by 2 to 4 times [1,2,3]. The American Diabetes Association (ADA) classifies DM into four generic types [4]. Type 1 DM (T1DM) is characterized by the destruction of the pancreatic β-cells by an unknown autoimmune mechanism, usually resulting in absolute insulin deficiency [5], and it also includes the latent autoimmune diabetes of adults (LADA). Type 2 DM (T2DM) is hallmarked by a progressive loss of adequate insulin secretion from the β-cells as the result of insulin resistance (IR) [6]. Special types of DM include those with monogenic causes [neonatal diabetes and MODY (mature-onset diabetes of the young)], diseases of the exocrine part of the pancreas (including cystic fibrosis, pancreatitis, etc.), drug- or chemical-induced DM (e.g., use of glucocorticoids), as well as DM induced after surgical operations (organ transplantation) [4]. Gestational DM (GDM) is the type diagnosed during the second or third trimester of pregnancy, averting post-gestation [4,7,8]. It is the most common complication of pregnancy and is associated with perinatal complications [8].

During the past decades, the global prevalence of T2DM and GDM has been increasing [9], as a result of environmental and lifestyle changes [10]. The most common complications in DM include diabetic ketoacidosis (DKA) and microvascular and macrovascular complications.

## 2. Ketones

**Figure 1:**
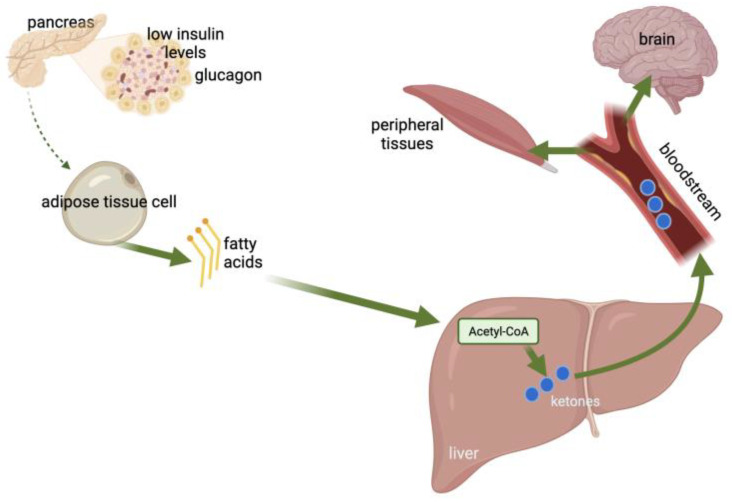
Ketogenesis (created with BioRender.com [17]).

In glucose-deprived conditions, ketone bodies are produced by the liver mitochondria through the catabolism of fatty acids [11] (Figure 1). Ketones are energy-rich metabolites and consist of acetone, 3-β-hydroxybutyrate (3BHB), and acetoacetate (AcAc) [12]. Fatty acids are used by the body as an alternative energy source and are oxidized to ketone bodies under insufficient glucose availability or starvation conditions [13]. The liver is the main organ producing ketone bodies, with fatty acids entering the mitochondria via carnitine palmitoyltransferase (CPT-1) and participating in β-oxidation for the production of acetyl-coenzyme A (acetyl-CoA) [14,15,16]. The produced ketone bodies are then transported extrahepatically for further oxidation [13] and used peripherally as an alternative energy source [11].

The reference range of total ketone body concentrations in healthy adults has a circadian rhythm and is approximately 100–250 µM [18]. However, in prolonged fasting and other conditions, the concentration of ketone bodies can rise to ~1 mM or even 20 mM [18]. Ketones are produced in the body under normal conditions, including during pregnancy and the neonatal period, when following a ketogenic diet (KD), fasting, or exercising [19]. Additionally, ketone synthesis is also augmented under pathological conditions, including cases of hyperemesis gravidarum, DKA, alcoholism, and a variety of metabolic disorders. Nonetheless, diet is the main regulator of total body ketone concentrations [12].

### 2.1. DKA-Induced Effects

It is widely known that DKA can cascade several adverse events and multiply the risk of developing diabetic complications [20]. In elevated concentrations, ketones lead to increased oxidative stress and inflammation and, through this mechanism, exert their harmful effects throughout the body. Cardiomyocytes, erythrocytes, and endothelial cells are the main cell types affected by oxidative stress [21]. Ketosis-induced oxidative stress is mediated by upregulation of nicotinamide adenine dinucleotide phosphate (NADPH) oxidases, activation of the mitogen-activated protein kinase (MAPK) pathway, and the nuclear factor kappa-light-chain enhancer of activated B cells (NF-κB). Vascular disease, atherosclerosis, and liver damage are direct consequences of this condition [21]. Additionally, elevated plasma ketone concentrations appear to be involved in reducing cell surface insulin receptors, leading to increased IR [20].

### 2.2. Elevated Ketone Concentrations during Pregnancy

Animal studies suggest that during pregnancy, increased ketone concentrations may induce harmful effects in the fetus and mother (Table 1) [22,23,24,25,26,27,28,29]. However, the exact pathophysiological mechanism behind these actions has not been fully elucidated [19]. Animal research has revealed alterations in embryonic organ growth following a maternal KD, associated with organ dysfunction in postnatal life [22]. Even a 24 h exposure to elevated ketone concentrations was shown to induce growth reduction and inhibition/delay of neural tube closure, depending on the embryo’s age and the ketone concentrations [23,28,29]. Interestingly, embryos also show a high adaptation capacity, with the ability to recover morphologically from the gross anatomical disturbance when elevated ketone concentrations are corrected and the recovery period is of sufficient duration [24,25,26]. Nonetheless, several histological alterations appear to remain in the affected tissues, without any adaptations being apparent [25].

### 2.3. Mild Ketonemia

On the other hand, in mild ketonemia, when ketone concentrations do not exceed a critical level, they seem to exert beneficial effects. Mild ketonemia is usually achieved after fasting or when following a KD. At low concentrations, ketone bodies act as signaling molecules and are involved in the post-translational modification of proteins. They regulate oxidative stress and inflammation and, through this mechanism, improve metabolic profiles and may extend a healthy lifespan. Due to these beneficial effects, they have become the target of research for their possible involvement in the treatment of various disorders, such as metabolic disorders, neurological and CVD disorders, and cancer [30].

## 3. Ketogenic Diets (KDs)

Since the time of Hippocrates, fasting has been traditionally used as a treatment for a variety of conditions [31]. As a therapeutic regime, fasting is the ancestor of today’s KD [31], with the latter mimicking the fasting state while altering the default metabolism towards the use of fats as the primary fuel source, produced through the catabolism of FFAs [32]. The first study of the use of fasting as a treatment was for epilepsy in 1911 by two French physicians, Guelpa and Marie [33]. In more detail, the effect of starvation was studied in 20 subjects (children and adults) suffering from epilepsy, and it was observed that the seizures were less severe during the diet period [33]. In the year 1921, the first observation was reported [34] that fasting is followed by the production of acetone and beta-hydroxybutyric acid in healthy individuals. The same result was observed after following a low-carbohydrate (CHO) diet (LCD), which was concomitantly high in fat content, known as the KD [35]. Therefore, the replacement of fasting with the KD was suggested, as they appeared to be equally effective, with the KD also having the ability to be applied for longer periods of time compared to fasting [33], with the latter often resulting in emaciation of the patients. Today, the KD is considered an effective therapeutic regime for drug-resistant epilepsy, as recommended by the Cochrane Collaboration and the National Institute of Healthcare and Excellence (NICE) [36,37]. In parallel, it has also shown possible benefits in a variety of neurological conditions, including mild cognitive impairment, cyclin-dependent kinase-like 5 (CDKL5) deficiency, pyruvate dehydrogenase complex deficiency, Alzheimer’s and Parkinson’s disease [38,39,40,41,42], and many more, gaining immense popularity.

### 3.1. Types of KDs

There is not one horizontal KD for all, but rather, various types of KDs exist based on the macronutrient contribution to the total energy intake (TEI), with some being “stricter” than the rest (Table 2). Overall, the KD reverses the typical dietary pyramid use of macronutrients, promoting a restrained CHO intake and a more liberal consumption of proteins and fats.

The classic KD is a strict diet, mathematically calculated for each person, and closely monitored [43]. It is high in lipids (90% of the TEI), but low in CHO and proteins. The medium-chain triglycerides (MCT) pattern is based on the fact that MCTs produce more ketones/g compared to long-chain triglycerides (LCT), allowing for more generous intakes of protein and CHO in the daily diet [44]. The MCT pattern was developed by Huttenlocher and associates [44]. The modified Atkins diet is more freestyle, allowing for a free intake of fat, protein, energy, and fluids, while limiting CHO intake. Finally, the low glycemic index (GI) [45] pattern was first suggested by Pfeifer and Thiele [46] following observations that adhering to the classic KD induced stability in the blood glucose levels, which was related to the mechanistic effects of the diet. It allows for some (10% of the TEI) CHO content based on low-GI [45] choices.

### 3.2. Advantages of the KD

The KD has recently emerged as an effective medical nutrition therapy (MNT) for overweight and obesity [47,48,49]. According to an umbrella systematic review [48], high-quality evidence supports the reduction in triglyceride concentrations, and moderate-quality evidence vouches for reductions in body weight, respiratory exchange ratio (RER), glycosylated hemoglobin (HbA1c), and increased total cholesterol levels. Furthermore, *N*-of-1 studies suggest that patients with overweight and obesity show preference for the KD over the typical hypocaloric diets [50].

As for other CVD risk factors often associated with overweight and T2DM, meta-analyses suggest that the KD also confers improvements in systolic and diastolic blood pressure [51] and does not appear to negatively affect kidney health, as indicated by stable blood–urea–nitrogen (BUN) and creatinine levels. Furthermore, the reduction in IR is closely linked to improved inflammatory status [52].

Nonetheless, the beneficial effects of KDs extend beyond their cardiovascular aspects. Research in mice has shown increased brown adipose tissue (BAT) levels following a KD [53], as a result of elevated ketone esters concentrations [54]. In fact, it is the greater postprandial lipid levels associated with the diet that increase the peroxisome proliferator-activated receptor α and γ transcription factors [55], initiating the transcription of uncoupling protein 1 and improving BAT differentiation [54]. In parallel, the thermogenic activity of the BAT is also increased [56].

Furthermore, the KDs have been shown to have appetite-suppressant effects, which may act as an important asset for improving adherence to low-energy diets and weight loss [57]. According to a systematic review [58], individuals adhering to energy-restricted KDs are less hungry and have a reduced desire to eat. The added clinical benefit of a KD is in preventing increases in appetite, irrespective of weight loss, as ketosis appears to provide a plausible anorexigenic mechanism [58], via the release of cholecystokinin (CCK), while reducing orexigenic signals [59].

Last, but not least, new research suggests circadian synchronicity following a KD and beneficial effects on sleep through the enhancement of slow-wave sleep and the rejuvenation of circadian programming [60].

### 3.3. Disadvantages of the KD

Despite its therapeutic potential, we still lack longitudinal data regarding the long-term effects of the KD. As a result, several researchers have highlighted the potential negative effects of long-term KD adherence regarding the primary and secondary prevention of CVD [61], based on the traditional atherogenic model. Recently, a large apparently healthy community-based cohort [62] associated elevated ketone bodies with a greater rate of CVD and mortality. Nonetheless, several studies have shown favorable results in the overall cardiometabolic profile of patients adhering to the KD, including weight loss, triglyceride levels, and high-density lipoprotein (HDL) cholesterol concentrations, as discussed in detail later. According to the National Lipid Association Nutrition and Lifestyle Task Force [63], the KD may confer advantages related to appetite suppression and a reduction in the concentrations of triglycerides and the required diabetes medication; however, it also demonstrates mixed effects on low-density lipoprotein (LDL) cholesterol levels. Moreover, we should also consider that the KD is the standard-of-care diet therapy for cystic fibrosis, followed by all patients, and no evidence of increased CVD risk has been shown to exist in this population [64]. Furthermore, a randomized controlled trial (RCT) [65] comparing the KD to a low-fat diet (both hypocaloric) among adult volunteers with overweight and hyperlipidemia motivated to lose weight for 6 months revealed that at the end of treatment, the KD changed the composition of LDL subclasses by increasing the proportion of large-sized buoyant LDL (with cardioprotective effects) and decreasing small-sized dense LDL (the primary cause of atherogenesis in the arterial intima). According to the American Heart Association [66], the KD has been shown to improve cardiovascular risk factors, including body weight, blood glucose, triglycerides, and HDL concentrations, when followed for a duration of 6 months. Nonetheless, it consists of a restrictive dietary pattern, raising concerns about nutrient adequacy [66].

Theoretically, another postulated limitation of the KDs is the low micronutrient content [67], posing a threat to the development of nutrient deficiencies. However, aside from thiamin deficiency, as most people on a KD tend to receive oral nutrient supplements (ONS) for micronutrients, no deficiencies have been noted in the literature thus far.

When initiating a KD, many individuals report “keto flu” symptoms, characterized by fatigue, headache, constipation, etc. [66]. These symptoms may act as deterrents to continuing the diet; however, they tend to improve over time as the body adapts to the alternative energy substrate [66]. Finally, the lack of food choices has also been noted, often leading to gradual non-adherence [66,68,69].

## 4. KDs for DM

Until the year 2019, the “healthy” diet for people with DM was a horizontal diet with ample CHO intake (50–55% of the TEI), adequate protein (15% of the TEI), and low fat (30–35% of the TEI). However, the 2019 consensus report published by the ADA [70] on MNT for adults with DM/prediabetes was a critical point in DM management, forming a paradigm shift in evidence-based dietetic practice.

Although the low-CHO diet (LCD) was first suggested as a treatment regime by Banting as early as 1864 [71], it was not until Dr Noakes was cleared for misconduct in 2016 that it started gaining awareness. Timothy Noakes, a professor in South Africa and patient with DM himself, was accused of misconduct for recommending adherence to a low-CHO eating pattern through social media. During the following years, a trial was conducted by the Health Professions Council of South Africa, during which scientific evidence was presented and appraised, and a plethora of scientists provided their opinion on the subject. This initiated the “diet wars”, with many scientists questioning the scientific rigor behind the nutrition guidelines, highlighting the need for an appraisal of the evidence and the formulation of more robust recommendations [72,73]. The increased scientific interest resulted in the production of several systematic reviews aiming to assess the efficacy of LCD in DM management [74,75].

### 4.1. The KD for T1DM

Before the discovery of insulin in 1920, the only treatment option for patients with T1DM was the LCD, with the CHO content of the diet restricted below 10 g/day [71]. After the introduction of insulin in the treatment of T1DM, the landscape changed dramatically, but diet remained an important contributor to the self-management of DM. The optimal treatment for T1DM is the combination of insulin with a healthy diet, aimed at limiting the consumption of CHO, since it is known that CHO is the main cause of postprandial hyperglycemia [76].

Table 3 details the existing primary research on the KD in patients with a T1DM diagnosis. Overall, it seems that when advice on following an LCD is given to patients with T1DM [77], better glycemic control is achieved by using less insulin each day (Table 3). Another study included 11 patients with T1DM and studied the effects of adhering to a KD (<55 g of CHO), indicating improvements in glycemic regulation and glycemic variability. However, these patients developed dyslipidemia and an increased risk for hypoglycemic events [78].

Some researchers argue that good compliance with the KD is difficult to achieve in T1DM, suggesting that patients often tend to give up after 1–2 years of implementation due to intolerance and difficulty in choosing foods [82]. Overall, the studies implementing a KD pattern in patients with T1DM, although somehow underpowered, reveal improvements regarding the HbA1c levels, the glycemic variability, and the use of insulin [77,78,79,80]. As for children, it has been suggested that children applying such strict diets may present developmental deficits, fatigue, and a long-term increased CVD risk [83]. However, growth is rather dependent on the amount of protein consumed and not the result of low CHO intake. Thus, more research is required to evaluate the effects of well-designed KDs on providing adequate protein intake in children with T1DM.

In addition to the involvement of diet in the treatment of T1DM, the literature has also referred to the involvement of diet in the pathogenesis of the disease. The normal functioning of the intestine is due to the delicate balance of the intestinal microbiota and the immunity of its mucosa. It has been found that when this balance is disturbed, various autoimmune diseases can develop, such as T1DM [84,85]. The intestinal microbiota normally includes *Clostridia*, microorganisms that produce butyrate and have immunomodulating functions. Butyrate has been shown to exert a protective effect on the development of pancreatic β-cell autoimmunity, and patients with T1DM have been found to have a low number of *Clostridia* [86,87,88]. It is widely known that the intestinal microbiome is influenced by dietary habits [85]. For example, a diet high in fat and salt intake, which is common in western countries, can induce IgA responses in the gut microbiome and lead to the production of autoantibodies [85]. On the other hand, there is also evidence in the literature that a high-fat diet, both in mice and in humans, is associated with a protective role against the development of autoimmunity due to a reduction in *Bacteroidetes* and an increase in *Firmicutes* [76]. In general, the gut microbiome influences autoimmunity, but the way has not yet been fully elucidated, and it is still unclear whether a KD and an LCD can confer prevention from the development of autoimmunity and delay the onset of T1DM.

### 4.2. The KD for T2DM

T2DM is the direct epiphenomenon of overweight and obesity and is initiated as a prediabetic state, hallmarked by IR. As part of the comprehensive treatment of T2DM with obesity, MNT aims to improve insulin sensitivity and reduce body weight. The current ADA guidelines recommend a reduction in total CHO intake in adults with T2DM, LCD, or very-LCD (VLCD) who fail to meet glycemic targets. However, no recommendations have been suggested for the “ideal” proportion of CHOs or fat to the TEI [70].

Today, numerous meta-analyses have shown efficacy of the KD in reducing body weight and other obesity indices (BMI and waist circumference) [47,51,89,90,91,92,93,94], improving HbA1c levels, and improving blood lipid profiles [47,51,89,90,91,92,93,94,95] (Table 4). The reduction in glucose-lowering medications has also been noted by many meta-analyses [75,89,91,93], with some scientists vouching that pharmacotherapy can not only be significantly reduced but even completely withdrawn [96], as shown by studies reporting DM remission (defined as HbA1c < 6.5%) post-adherence to a KD [93,97]. According to Goldberg [93], patients adhering to an LCD for a period of 6 months may experience DM remission without adverse consequences. Moreover, greater CHO restriction is associated with an enhanced glucose-lowering effect [75].

In an early meta-analysis, Snorgaard and colleagues [75] suggested that the positive effects of the KD only last for approximately a year, with HbA1c being similar between control and KD thereafter. As with T1DM, low adherence was one of the issues reported by trialists [91], with low adherence to the strict dietary regime being one [89]. In fact, the more strict regime followed at VLCDs appears to be less effective than the less restrictive LCDs for improving body weight as a result of lower diet adherence [93].

**Table 4:** Systematic reviews assessing the effect of KDs in T2DM/prediabetes.

First Author	Included Studies	Results
Choi [47]	14 RCTs	The effects of KD on glycemic control were greater relative to those of LFD for patients with T2DM, indicated by lower HbA1c and HOMA, while comparable effects were observed for nondiabetic patients. KDs led to substantial BW loss, irrespective of patients’ DM status at baseline and improved lipid profiles in terms of lower TG and greater HDL for patients with DM. KDs were more effective in improving metabolic parameters associated with glycemic, BW and lipid control in patients with overweight/obesity, and especially preexisting DM, as compared to LFDs.
Goldenberg [93]	23 RCTs	At 6 months, LCDs achieved higher rates of diabetes remission compared with control diets. Large clinically important improvements were seen in BW loss, TG, and insulin sensitivity at 6 months, though they were diminished at 12 months. VLCDs were less effective than less restrictive LCDs for BW loss at 6 months, but this was explained by diet adherence.
Luo [94]	21 RCTs	LCD exerted a greater impact on CV risk factors in overweight/obese patients with T2DM, with lower FPG and HbA1c levels. LCD reduced BMI, BW, and WC in overweight/obese patients with T2DM. Also, adherence to KDs improved lipid profiles with TG concentrations being lowered and HDL noting an upward trend.
Parry-Strong [95]	8 RCTs of ≥6 months duration	A VLCD/KD may cause reductions in HbA1c and TG levels in patients with pre-diabetes/T2DM, but evidence of an advantage over other strategies remains limited.
Rafiullah [91]	8 RCTs	Compared with control diets, the VLCD resulted in a greater decrease in HbA1c and BW loss after 3 and 6 months. The VLCD was not better than a control diet after 12 months. It was superior in decreasing TG, increasing HDL and reducing the use of antidiabetic drugs for up to 12 months.
Snorgaard [75]	10 RCTs	In the first year of intervention, LCD was followed reduced HbA1c more compared with HCD. The greater the CHO restriction, the greater the glucose-lowering effect. At 1 year or later however, HbA1c was similar between the 2 diet arms. The effect of the 2 diets on BMI/BW, LDL, QoL, and attrition rate was similar throughout interventions.
Tinguely [89]	14 CTs	KD improves HbA1c at 3 weeks, and the effect persists for at least a year, a result associated with a reduction in glucose-lowering medications. Additionally, the short-term observed BW loss is maintained with a long-term diet. Adequate support (psychological counseling, enhancing positive affectivity, reinforcing mindful eating) is required to achieve benefits and ensure adherence.
Yuan [90]	13 RCTs	Post-KD, the levels of fasting glucose, HbA1c, total cholesterol, LDL and TG decreased, but HDL increased. In addition, BW, WC, and BMI also decreased.
Zaki [92]	15 RCTs and observational studies	An LCD induced a greater reduction in the HbA1c than other diets. A decrease in HbA1c and BW was recorded when the KD was consumed compared to the control diets.
Zhou [51]	8 RCTs	The KD reduced BW, WC, HbA1c and TG, and increased HDL levels. The KD may be an effective dietary intervention for BW, glycemia and lipid profiles in overweight with T2DM.

BMI, body mass index; BW, body weight; CT, clinical trial; CV, cardiovascular; DM, diabetes mellitus; FPG, fasting plasma glucose; HbA1c, glycosylated hemoglobin; HCD, high carbohydrate diet; HDL, high-density lipoprotein; HOMA, homeostatic model assessment index; KD, ketogenic diet; LDL, low-density lipoprotein; LFD, low-fat diet; QoL, quality of life; RCT, randomized controlled trial; T2DM, type 2 diabetes mellitus; TG, triglycerides; VLCD, very low carbohydrate diet; WC, waist circumference.

## 5. Assessing the Concentration of Ketone Bodies

In the human body, ketone bodies can be quantified in the capillary blood, serum, and urine. Urine sticks used to measure ketones qualitatively and semi-quantitatively determine AcAc and acetone. They cannot, however, identify β-hydroxybutyrate (BHB). The urine sticks are impregnated with a nitroprusside reagent, which reacts with AcAc and acetone and, in this way, determines their existence or not. Because during DKA, AcAc is converted to hydroxybutyrate, AcAc levels are reduced, and its concentration in urine is also reduced. As a result, the assessment of urine ketone levels during DKA can often be misleading, underestimating the severity of acidosis. Furthermore, since urine is not expelled from the bladder immediately and may be retained for some time, measuring urine ketones is not always representative of what is happening simultaneously in the body and cannot determine the changes that occur in real time. In addition, the patient is not always ready to give a urine sample, especially in cases of ketosis characterized by dehydration. When capillary BHB and urinary ketone testing were compared, the first was shown to have high sensitivity, specificity, positive predictive value, and negative predictive value in identifying DKA [98]. As a result, the assessment of ketones in the urine is not the most preferred method when an immediate result is required [98]. Moreover, in patients with T1DM point of care (POC), blood ketone monitors have been shown to exert benefits in reducing assessment time, the duration of the admission, and the time until recovery from DKA [98,99]. Table 5 details all of the technology used to assess ketone body levels and diagnose DKA.

For all these reasons, the assessment of blood ketone levels is preferred since it is considered more reliable. Recently, the validity and reliability of measuring ketone bodies, and specifically BHB in capillary blood as bedside testing, have begun to be supported. This method seems to provide reliable results and has the advantage of immediate assessment and intervention. The approved POC ketone meters assess capillary blood ketone using dry-chemistry methodology, and they have been compared with reference enzymatic spectrophotometric assays and found to be valid [105].

A recent prospective study [106] included 171 patients with hyperglycemia (>11 mmol/L) and capillary blood ketone > 0.1 mmol/L presented at the emergency department. Urine, serum, and capillary ketones were measured in all patients. Some of the patients were diagnosed with ketoacidosis, and some with ketonemia alone. Capillary blood ketone concentrations showed greater sensitivity and specificity for the diagnosis of DKA compared with the level of urine ketone bodies. Akin capillary and serum ketone concentrations were noted [106]. In conclusion, it was shown that measuring ketones in capillary blood could be a good alternative to serum ketones assessment, and is certainly superior to measuring ketones in the urine [106].

In addition to blood and urine, breath acetone concentration is another non-invasive measure of ketosis [107]. The method relies on headspace solid-phase microextraction and gas chromatography-mass spectrometry (HS-SPME/GC-MS), with breath acetone concentrations varying between 1 and 1250 ppm in a healthy non-fasting state and DKA, respectively [108]. When compared to capillary blood glucose and ketone levels (BHB and AcAc), strong relationships were observed [107] in patients with T2DM. Furthermore, when tested on patients with T1DM, the breath ketone analyzer showed good sensitivity and low specificity to detect ketosis in adults but not in children [100] (Table 5).

Recently, the use of saliva enzymatic sensor strips has also been proposed as an alternative non-invasive method for detecting DKA and assessing ketone levels for lifestyle factors; however, no research has yet evaluated this method [101].

### 5.1. Ketonemia and Ketonuria in T1DM

In patients with T1DM, elevated glucose concentrations lead to higher circulating ketone levels and are associated with complications. There is a difference, however, between the ketogenesis induced by fasting, or KD, and the ketogenesis induced by uncontrolled DM [109]. During fasting or when adhering to a KD, the increased production of ketone bodies is intended to function as an alternative source of energy. On the contrary, the production of ketones caused by uncontrolled DM is the result of dysregulated metabolism and a lack of insulin and is not intended to function as an energy source [110]. Insulin contributes to the clearance of ketone bodies, and its deficiency leads to reduced clearance. Additionally, in patients with DM, the activity of BHB dehydrogenase is also reduced, a fact that also leads to an increase in the total concentration of ketones in the body [20].

Insulin inhibits lipolysis and increases the utilization of ketone bodies from peripheral tissues. Insulin also inhibits hormone-sensitive lipase (HSL) activity, which releases FFAs into the circulation. In T1DM, where insulin deficiency is apparent, an increase in lipolysis is observed [111]. Thus, in insulin deficiency, glucagon prevails, activating carnitine acyltransferase (CAT1), by which the FFAs are transferred from the circulation to the mitochondria of the liver cells. Then, the oxidation of FFAs takes place, and acetyl-CoA is produced [112]. Acetyl-CoA is used for the synthesis of ketone bodies (AcAc and BHB) [113].

DKA is the most frequent complication in T1DM, characterized by hyperglycemia, acidosis, and ketosis [114]. When the levels of counter-regulatory hormones are increased, the activation of HSL in the adipose tissue leads to increased triglyceride catabolism and the production of non-esterified fatty acids and glycerol. Glycerol is used as a substrate for gluconeogenesis, and fatty acids are oxidized to ketone bodies in the liver. In parallel, the increased concentration of glucagon leads to higher carnitine concentrations and decreased hepatic malonyl CoA in the liver, which stimulates CAT1, a key enzyme in the ketogenesis process, resulting in increased ketone body concentration. Moreover, in DKA, reduced ketone body clearance is observed due to insulin deficiency and decreased peripheral glucose utilization. DKA manifests quickly, and its clinical signs include polyuria, polydipsia, vomiting, abdominal pain, dehydration, weakness, tachycardia, kussmaul respiration, and, in severe cases, altered mental status [115].

### 5.2. Ketonemia and Ketonuria in T2DM

T2DM is a metabolic disease and is mainly characterized by IR and the coexistence of partial β-cell dysfunction. The incidence of the disease has increased rapidly in the last decades worldwide due to the increase in obesity and the sedentary lifestyle [116]. It has been estimated that in 2019, 463 million adults suffered from T2DM [117].

During the day, there is variation in the concentration of ketone bodies in the human body. Usually, ketone concentrations increase around midnight and in the morning hours (after fasting) and tend to decrease during the day after consuming CHO-rich meals. In parallel, as the age of patients increases, the ketone levels tend to decrease, in particular during the pre-dinner state [118].

### 5.3. Ketonemia and Ketonuria in Ketosis-Prone DM (KPDM)

Although in the past, a strict classification between different types of DM existed, it has since been observed that atypical forms occur more frequently. More specifically, a new subtype of DM has been observed characterized by DKA in patients lacking the typical phenotype of autoimmune T1DM [119]. This syndrome is characterized as “ketosis-prone DM” (KPDM) [120]. The first cases of KPDM were described in the 1960s in Africa, and generally, non-Caucasian individuals without a T1DM diagnosis are more prone to this type of DM [121]. It was observed that patients who presented with ketosis and required insulin therapy over time experienced remission of DM and were able to be released from insulin therapy, something that does not happen in patients with T1DM [122]. An increase in the records of such heterogeneous incidents followed in many nationalities. Great heterogeneity was observed among the described cases, so in the early 2000s, an attempt was made to classify them into subcategories, aiming to better understand and manage this distinct condition. The classification was based on the presence of islet autoantibodies and β-cell function reserves. As a result, a systematic classification of KPDM into four subcategories was established in 2003 using the Aβ classification system [123], as detailed in Table 6.

The most common form of KPDM is the A-β+KPDM, affecting about 50% of KPDM cases, followed by the types A-β-KPDM and A+β-KPDM at a prevalence ratio of 20% each, while the rarest form is the A+β+, which only affects 10% of cases [123].

Patients with KPDM and absence of β-cell function, A+β- and A-β- are diagnosed with DM early on, at a young age. They usually have a low body weight, and their treatment requires the administration of insulin. In the vast majority of patients, when there is loss of β-cell function and patients have been classified in the β-subcategory, the condition is considered irreversible, but in very few cases, at a rate of <1%, some improvement in β-cell function may be observed. The A+β- subgroup essentially corresponds to the autoimmune T1DM [123]. Subgroups A-β- and A+β- have similar phenotypes, and their notable difference is the presence or absence of islet autoantibodies. Patients who retain some functionality in the β cell, i.e., A-β+ form, have clinical features similar to T2DM but present with DKA. After the treatment of DKA, β-cell functionality remains and improves over time, and as a result, insulin dependence recedes for most of these patients within 4 to 8 months, and they achieve good glycemic control on oral medications for the following 4 years, at the very least. About half of these patients manage to maintain adequate β cell function for many years, and they achieve satisfactory glycemic control only on oral antidiabetic tablets. The other half, however, rapidly loses β-cell functionality and, after a short period on oral antidiabetic tablets, returns to insulin dependence again. It appears that patients diagnosed with DKA triggered by a stressful event have a worse prognosis and are more likely to be insulin dependent than those diagnosed with unprovoked DKA. The unprovoked A-β+ subgroup is characterized by late onset, presents more often in men with increased body weight, DKA at the initial diagnosis of diabetes, and the absence of islet autoantigens. In contrast, the provoked A-β+ subgroup presents in both males and females with a history of T2DM [124]. Patients with the A+β+ form of the disease share similar clinical features as those with T2DM, but also exhibit islet autoimmunity and usually present DKA at the diagnosis of diabetes. And in this form, about half of the patients will rapidly lose β-cell function and become insulin-dependent, while the other half have a better prognosis with prolonged preserved β-cell function and are insulin-independent [125].

With regard to the management of these patients post-diagnosis, the initial approach involves inpatient management of DKA with intravenous fluids and insulin. Subsequently, these patients are discharged from the hospital with instructions for subcutaneous insulin use and recommendations for the follow-up examination [126].

### 5.4. Ketonemia and Ketonuria in GDM

During pregnancy, many changes occur in the mother’s body, aiming to accommodate the developing fetus. Early pregnancy is characterized by anabolic processes, while late pregnancy is a catabolic phase [127]. In the second and third trimesters, an increase in lipolysis is observed, which leads to the production of FFAs, which are used as an energy source for the mother, while the fetus uses mostly glucose. As a result, during pregnancy, there is increased ketogenesis. Cases of ketoacidosis have been reported, even among healthy pregnant women, during periods of starvation. Moreover, women with GDM have been shown to have a higher ketone metabolism than those without GDM and are thus more prone to the development of ketonemia [19,128]. This may be the result of impaired pancreatic β-cell function, restricting insulin secretion while impairing glucose tolerance [129]. For this reason, women with GDM are encouraged to restrict their CHO intake and divide the intake of CHO throughout the day in order to achieve better glycemic regulation [130,131]. Nonetheless, this can often result in the development of ketonuria. Ketonuria is also observed in women with GDM, especially after fasting, and is correlated with elevated blood BHB concentrations [130]. However, it should be noted that, at the moment, we have not yet defined the level at which CHO restriction leads to increased ketone levels [132]. Few studies have assessed the effect of CHO restriction on the concentration of ketones in women with GDM (Table 7).

It appears that some women exhibit urinary ketones [136] even when consuming the threshold value of 175 g/day suggested by the scientific authorities [131,137]. On the other hand, in the study conducted by Mijatovic [134], a mean intake of 165 g of CHO/day was not associated with detectable urinary ketone levels in women with GDM. Similarly, Potter and associates [135] failed to reveal differences in the urinary ketone levels between women with GDM on an LCD compared to untreated ones. Based on these findings, several scientists are advocating for lowering the dietary CHO threshold for managing GDM [138], whereas others are suggesting considering the placental CHO needs and increasing the threshold to 220 g of CHO/day [139].

Of note, aside from the duration of pregnancy, ketosis is also observed during labor as a residue of the increased physical stress and is linked with a greater likelihood of augmentation of labor, forceps-assisted delivery, and postpartum hemorrhage. Ketonuria has been related to prolonged labor duration [140].

It has been proven that ketone bodies cross the placenta. Recently, a radioisotope-labeled BHB was administered to pregnant rats, and within 5 min, it was detected in the fetus’ plasma [141]. During the third trimester, increased plasma 3HB has been associated with impaired neuropsychological development in the offspring [142]. Animal and in vitro studies [19] have revealed that ketone body levels are influencing cardiomyocyte glucose uptake [143], embryonic brain structural development [144], as well as tubular cell growth [145]. In an early study, ketonuria during pregnancy was associated with reduced intellectual status of the offspring at the age of 5 years, and reduced birth weight was also related to impaired intellectual status at the ages of 3 and 5 years [146]. However, no other data have verified this observation [147]. In parallel, data linking hyperketonemia or hyperketonuria with perinatal adverse events remain limited, and we are also lacking recommendations to manage such cases. As a result, the management of pregnant women with hyperketonemia/hyperketonuria has not been determined.

### 5.5. Ketone Bodies and Antidiabetic Medication

The main antidiabetic medications that have been linked to an increase in ketone body concentrations are sodium-glucose transport protein 2 (SGLT-2) inhibitors. These drugs exert their actions on the kidneys, where they increase the reabsorption of ketone bodies, and they also act on the pancreas, where they stimulate the secretion of glucagon from α-cells, resulting in an increase in ketone production and a simultaneous decrease in insulin production [148]. As a result, the use of CHO decreases and lipοlysis is amplified, leading to a further increase in the production of ketones in the liver [149]. This increase in ketone body concentrations among patients on SGLT-2 inhibitor therapy can rarely even lead to DKA, even when normal blood glucose concentrations are recorded [150]. Moreover, a recent study indicated that the co-administration of pioglitazone with SGLT-2 inhibitors may independently increase blood ketone body concentration [151].

On the other hand, liraglutide appears to exert an inhibitory effect on ketogenesis. In a recent trial [152], 26 patients with uncontrolled T1DM were randomized to liraglutide injections or placebo, respectively, after fasting, and it was shown that liraglutide did not induce any increments in ketone body concentrations in contrast to placebo. The action of liraglutide in the suppression of ketogenesis seems to be attributed mainly to the reduction in glucagon, but it is possible that liraglutide also exerts a direct action in the hepatocytes [152].

Regarding metformin, the data in the literature are conflicting. On the one hand, metformin seems to act antagonistically on BHB, tampering down the inflammation propelled by the increased BHB concentrations [153]. On the other hand, metformin also seems to increase the oxidation of fatty acids, and animal studies revealed an increase in ketone body concentration following the administration of metformin [154].

Last but not least, insulin is a well-studied drug, and it is widely known that its deficiency causes ketogenesis, while insulin administration reduces the concentrations of plasma ketone bodies [155]. However, it is worth noting that by administering insulin, initially, an increase in urine ketone levels is observed, and during the initial phase of recovery from DKA, BHB is converted to AcAc, which is the main ketone body assessed in the urine [156].

## 6. Conclusions

In conclusion, it seems that ketone bodies are involved in both the diagnosis and the lifestyle treatment of DM types, as well as in the diagnosis of DM complications. Early ketone detection using POC technologies can aid in the early diagnosis of DKA. Regarding the assessment of ketones in the blood and urine of patients with DM, more research is required to interpret the results and use this knowledge for the benefit of the patients. Specific medications have been shown to interact with ketones, altering the physiological response. Last but not least, although the KD appears to be a promising new addition to the MNT regimes for managing DM, further research is warranted to support its adoption.

## Figures and Tables

**Table 1:** In vitro and in vivo animal studies assessing possible effects of elevated ketone levels in pregnant women.

Sample	Intervention(s)/ Exposure(s)	Results	First Author
Mouse embryos of two distinct stages (3–4 and 5–6 somites)	Racemic mixture of DL-BHB at levels of 8, 16, or 32 mM/L (24 h)	Growth reduction and inhibition/delay of neural tube closure were noted in the cranial and/or caudal regions of exposed embryos. The effects were dose- and age-dependent, with younger embryos being more affected, and higher doses producing greater malformations. Cytoplasmic vacuoles in the neuroepithelium, mesenchyme and ectoderm were noted, involving mitochondria undergone high-amplitude swelling with matrix density loss and cristae.	Horton [23]
Early mouse somite embryos	Culture for 4, 8, or 24 h in the presence of 32 mM DL-BHB and then culture (24 h) in control serum	Treated embryos showed progressive mitochondrial alterations, starting at 4 h with loss of matrix density, culminating at 24 h with high-amplitude swelling, complete matrix density loss and cristae disappearance. These changes were reversible following removal from BHB and culturing for 24 h in control serum. The early somite embryos showed a limited capacity to oxidatively metabolize BHB.	Horton [24]
Whole embryo cultures	24 h culture in: (i) Δ-BHB (48 mM), or (ii) control medium	All embryos exhibited NTD and lower rates of glucose metabolism by the PPP and Krebs cycle, compared to controls. The effect of the Δ-isomer on the Krebs cycle may result from glucose intermediates replacement generated from D-BHB metabolism.	Hunter [27]
Rat embryos at 9.5 days of gestation	Cultured in vitro for 24/48 h, with/without 4 × 10(-2) M BHB for all, or part of the culture period	Embryos exposed to BHB for a complete 48 h culture were more affected than those exposed for part of the culture and embryos were more vulnerable to BHB during the first ½ of a 48 h culture than during the second ½. Embryos cultured with BHB from 9.5 days of gestation for 24 h revealed some BHB effects after 24 h in culture. Many abnormalities were embryonic retardations, with embryos showing characteristics of normal, yet younger embryos.	Moore [29]
Early somite stage mouse embryos	Culture in: (i) control serum (60 h) (ii) serum with 32 mmol/L DL-BHB (24 h), followed by control serum (36 h, recovery) (iii) 32 mmol/L DL-BHB serum (60 h)	Although neural tube closure occurred in the recovery arm, complete recovery was limited to the ventral regions of the forebrain. The remainder of the prosencephalon and the rhombencephalon failed to catch-up growth completely. In these areas, cell numbers were approximately 70% of control values. Although the gross anatomical disturbances produced by high ketone levels may be compensated for, several histological alterations remain.	Shum [25]
Early somite stage mouse embryos	32 mM DL-BHB (24 h, Period I), and then transfer in control medium (36 h maximum, Period II)	At the end of Period I, all D,L,-BHB-exposed embryos were growth-retarded with NTD regarding closure. At 36 h of Period II, cranial and caudal NTD of embryos were reduced. These embryos also exhibited an excess in growth velocity during recovery thus, at the end of Period II, total protein content was comparable to control. Embryos who did not enter the control serum remained growth-retarded and showed more cranial and caudal NTD.	Shum [26]
CD-1 mouse embryos whose mothers were fed either an SD or a KD	SD or KD, 30 days prior to, as well as during gestation	At E13.5 the average KD embryo was volumetrically larger, with a larger heart but smaller brain, pharynx, hypothalamus, midbrain, cervical spinal cord and pons, compared with the SD embryo. At E17.5, KD embryos were smaller, with smaller hearts and thymuses, but with enlarged cervical spines, midbrains, thalamus, and pons.	Sussman [22]
in vivo embryos of control or streptozotocin-diabetic rats at gestational days 9–11	Cultured in a whole-embryo culture system for 48 h with high concentration of DM-related substrates and metabolites	Cytoplasmic vacuoles were observed in the ectoderm of day-9 embryos and in the neuroepithelium and blood cells of days-10–11 embryos of diabetic rats. These were mitochondria undergoing large-amplitude swelling with matrix density loss and disturbed cristae. No differences were noted in the brain, heart, or liver of day-15 fetuses from normal and diabetic rats. Day-9 embryos cultured in high concentrations of D-glucose, Pyr, BHB, and α-KIC for 48 h also showed high-amplitude mitochondrial swelling in the neuroepithelium.	Yang [28]

α-KIC, α-ketoisocaproate; BHB, β-hydroxybutyrate; DM, diabetes mellitus; E13.5, embryonic days 13.5; E17.5, embryonic days 17.5; KD, ketogenic diet; NTD, neural tube defects; PPP, pentose phosphate pathway; Pyr, pyruvate; SD, standard diet.

**Table 2:** Distinct KD patterns.

KD Type	Fat (% TEI)	Protein (% TEI)	CHO (% TEI)
Classical	90%	10% (protein + CHO)	
MCT	75% (21–25% from LCT and 45–55% from MCT)	25% (protein + CHO)	
Modified Atkins	nearly 75%	liberal intake	10–20 g/day
Low GI	60%	30%	≈10% (40–60 g/day with a low GI <50)

CHO, carbohydrate; GI, glycemic index; KD, ketogenic diet; LCT, long-chain triglycerides; MCT, medium-chain triglycerides; TEI, total energy intake.

**Table 3:** Studies implementing KD patterns in adult patients with T1DM.

First Author	Design	Participants	Intervention(s)	Duration	Results
Krebs [77]	RCT	N = 10 patients	(i) typical CHO counting course (ii) same course + advice on following an LCD (75 g/day)	12 weeks	The CHO-restricted arm showed reductions in HbA1c and insulin use and non-significant reductions in BW. No changes in BP, creatinine or lipid levels were noted, and all outcomes in the CHO-counting arm remained unchanged. No change was noted in the glycemic variability.
Leow [78]	Observational cohort	N = 11 patients	KD (<55 g CHO/day)	2.6 ± 3.3 years	KD resulted in excellent HbA1c levels and low glycemic variability, but may also be associated with dyslipidemia and a high incidence of hypoglycemic episodes.
Ranjan [79]	OL cross-over RCT	N = 10 adults on IP	(i) isocaloric HCD (≥250 g/d) (ii) isocaloric LCD (≤50 g/d)	1 week each intervention	Diet adherence was high and glucose levels were similar in both diets. The LCD resulted in more time with glucose between 3.9 and 10.0 mmol/L, less time with values ≤ 3.9 mmol/L, and less glucose variability than the HCD. CV markers were unaffected, but glucagon, FFA, and ketone concentrations were higher post-LCD.
Schmidt [80]	OL cross-over RCT	N = 14 patients with sensor-augmented IPs	(i) LCD < 100 g CHO/d) (ii) HCD > 250 g CHO/d)	12 weeks each intervention	Time spent in the range 3.9–10.0 mmol/L did not differ, but time at <3.9 mmol/L and glycemic variability were lower at LCD. No severe hypoglycemia events were recorded. LCD induced a BW loss (2.0 ± 2.1 kg) and HCD a BW gain (2.6 ± 1.8 kg), but no other CV risk factors were affected.
Nolan [81]	Case study	N = 1 cyclist who successfully undertook a 4011 km cycle across Australia	VLCD	20 days	Remarkable glycemic stability was noted, with 80.4% of time spent at 3.9–10 mmol/L. Interstitial glucose was <3 mmol/L for 2.1% of this time, and only one episode of hypoglycemia was recorded.

BP, blood pressure; BW, body weight; CHO, carbohydrate; CV, cardiovascular; FFAs, free fatty acids; HbA1c, glycosylated hemoglobin; HCD, high-carbohydrate diet; IP, insulin pump; KD, ketogenic diet; LCD, low carbohydrate diet; OL, open-label; RCT, randomized controlled trial; T1DM, type 1 diabetes mellitus; VLCD, very low carbohydrate diet.

**Table 5:** Technology used to assess ketone bodies levels and diagnose DKA *.

	Assay	Ketones Assessed	Cut-Off Used (If Any)	Sensitivity (%)	Specificity (%)	Target Population	Reference
Breath	HS-SPME/ GC-MS	Acetone	3.9 ppm	94.7	54.2	Adults with T1DM	[100]
Saliva	Enzymatic sensor strip	BHB	NR	NR	NR	NR	[101]
Capillary blood	Dry chemistry	BHB	>3 mmol/L	100	89	Adults	[102]
				90.4	100	Children with T1DM	[103]
Urine	Semiquantitative assay	AcAc	≥2+	89.9	52.7	T2DM	[104]
				84.9	86.5	Children with T1DM	[103]

AcAc, acetoacetate; BHB, β-hydroxybutyrate; DKA, diabetic ketoacidosis; GC-MS, gas chromatography-mass spectrometry; HS-SPME, headspace solid-phase microextraction; MEM, manual enzymatic method; NR, not reported; ppm, part per million; T1DM, type 1 diabetes mellitus; T2DM, type 2 diabetes mellitus; * the gold standard is plasma MEM.

**Table 6:** KPDM subtypes according to the Aβ classification system [123].

(i)	A+β-KPDM:	Characterized by the presence of islet autoantibodies and absence of β cell function
(ii)	A+β+KPDM:	Characterized by the presence of islet autoantibodies with preserved β cell functional reserve
(iii)	A-β-KPDM:	Characterized by absence of islet autoantibodies with absence of β cell function
(iv)	A-β+KPDM:	Characterized by absence of islet autoantibodies with preserved β cell functional reserve

KPDM, ketosis-prone diabetes mellitus.

**Table 7:** Primary studies evaluating the effect of CHO restriction in the concentration of ketones, in women with GDM.

First Author	Participants	Design	Interventions	Results
Major [133]	*n* = 21 women with GDM on an LCD (CHO < 42% of TEI) *n* = 21 women with GDM on HCD (CHO > 45% of TEI)	CC	N/A	Reductions in postprandial glucose values were observed and fewer subjects required insulin for glycemia in the LCD arm. The incidence of LGA infants, cesarean deliveries for cephalopelvic disproportion and macrosomia were lower in the LCD arm. Urinary ketones were only identified in 2 women, both following an LCD, clearing their urinary ketones when CHO intake was increased.
Mijatovic [134]	N = 46 women with GDM	RCT	(i) modest LCD (∼135 g of CHO/day) for 6 weeks (ii) routine care (∼200 g of CHO/day) for 6 weeks	No detectable differences were apparent in blood ketones between LCD (mean intake of 165 g of CHO/day) and routine care arm, although CHO and TEI were lower in the intervention arm. No differences were noted regarding birth weight, rate of LGA infants, % fat mass, or FFM between groups. A modest LCD does not result in increased fasting BHB levels.
Potter [135]	*n* = 7 non-diabetic women (A) *n* = 7 women with mild GDM at diagnosis (B), and *n* = 7 women with mild GDM post-treatment with a 150 g CHO diet (C)	CC	N/A	Glucose levels were indifferent between groups. Ketone body levels were elevated in the GDM group prior to treatment (B) and rose higher after treatment with the 150 g CHO diet (C). Lactate levels were reduced when on the restricted CHO diet.
Tsirou [136]	N = 43 women with GDM	CT	(i) VLED (175 g of CHO/day) (ii) VLED + exercise (175 g of CHO/day) (iii) LED (175 g of CHO/day) (iv) LED + exercise (175 g of CHO/day)	GWG was lower in the VLED and higher in the LED arms. No differences were noted in the type of delivery, birth weight, composite score, prematurity, depression, RQ, Apgar score, MUAC, or insulin use. Most infants (88.4%) were AGA, born at a gestational age of 37–42 weeks (95.3%). Only 9.3% of the mothers experienced delivery complications, with the majority being at the VLED + exercise arm. The composite score was low (range 0–2.5) for all, indicating a “risk-free” pregnancy outcome. No differences were noted in the urine ketone levels between groups.

AGA, appropriate-for-gestational age; BHB, β-hydroxybutyrate; CC, case control; CHO, carbohydrate; CT, clinical trial; FFM, fat-free mass; GDM, gestational diabetes mellitus; GWG, gestational weight gain; HCD, high-carbohydrate diet; LCD, low-carbohydrate diet; LED, low-energy diet (1800 kcal/day); LGA, large-for-gestational age; MUAC, middle-upper arm circumference; N/A, not applicable; RCT, randomized controlled trial; RQ, respiratory quotient; TEI, total energy intake; VLED, very low-energy diet (1800 kcal/day).

## Data Availability

All data are available within the manuscript text.

## Abbreviations

3BHB	3-β-hydroxybutyrate
α-KIC	α-ketoisocaproate
AcAc	Acetoacetate
Acetyl-CoA	Acetyl-coenzyme A
ADA	American Diabetes Association
AGA	Appropriate-for-gestational age
BAT	Brown adipose tissue
BHB	β-hydroxybutyrate
BMI	Body mass index
BUN	Blood–urea–nitrogen
BW	Body weight
CAT1	Carnitine acyltransferase 1
CC	Case-control
CCK	Cholecystokinin
CDKL5	Cyclin-dependent kinase-like 5
CHO	Carbohydrate
CPT-1	Carnitine palmitoyltransferase
CT	Clinical trial
CV	Cardiovascular
CVD	Cardiovascular disease
DKA	Diabetic ketoacidosis
DM	Diabetes mellitus
FFAs	Free-fatty acids
FFM	Fat-free mass
FPG	Fasting plasma glucose
GC-MS	Gas chromatography-mass spectrometry
GDM	Gestational diabetes mellitus
GI	Glycemic index
GWG	Gestational weight gain
HbA1c	Glycosylated haemoglobin
HCD	High-carbohydrate diet
HDL	High-density lipoprotein
HOMA	Homeostatic model assessment index
HSL	Hormone-sensitive lipase
HS-SPME	Headspace solid-phase microextraction
IP	Insulin pump
IR	Insulin resistance
KD	Ketogenic diet
KPDM	Ketosis-prone diabetes mellitus
LADA	Latent autoimmune diabetes of adults
LCD	Low-carbohydrate diet
LCT	Long-chain triglycerides
LDL	Low-density lipoprotein
LED	Low-energy diet
LFD	low-fat diet
LGA	Large-for-gestational age
MAPK	Mitogen-activated protein kinase
MCT	Medium-chain triglycerides
MEM	Manual enzymatic method
MNT	Medical nutrition therapy
MODY	Mature-onset diabetes of the young
MUAC	Middle-upper arm circumference
N/A	Not applicable
NADPH	Nicotinamide adenine dinucleotide phosphate
NICE	National Institute of Healthcare and Excellence
NF-κB	Nuclear factor kappa-light-chain enhancer of activated B cells
NTD	Neural tube defects
NR	Not reported
OL	Open label
ONS	Oral nutrient supplements
POC	Point of care
PPP	pentose phosphate pathway
Pyr	Pyruvate
QoL	Quality of life
RCT	Randomized controlled trial
RQ	Respiratory quotient
SD	Standard diet
SGLT-2	Sodium-glucose transport protein 2
T1DM	Type 1 diabetes mellitus
T2DM	Type 2 diabetes mellitus
TEI	Total energy intake
TG	Triglycerides
VLCD	Very low carbohydrate diet
VLED	Very low-energy diet
WC	Waist circumference
